# The adaptor protein DCAF7 mediates the interaction of the adenovirus E1A oncoprotein with the protein kinases DYRK1A and HIPK2

**DOI:** 10.1038/srep28241

**Published:** 2016-06-16

**Authors:** Florian Glenewinkel, Michael J. Cohen, Cason R. King, Sophie Kaspar, Simone Bamberg-Lemper, Joe S. Mymryk, Walter Becker

**Affiliations:** 1 Institute of Pharmacology and Toxicology, RWTH Aachen University, Aachen, Germany; 2 Departments of Microbiology & Immunology and Oncology, University of Western Ontario, London, Ontario, Canada

## Abstract

DYRK1A is a constitutively active protein kinase that has a critical role in growth and development which functions by regulating cell proliferation, differentiation and survival. DCAF7 (also termed WDR68 or HAN11) is a cellular binding partner of DYRK1A and also regulates signalling by the protein kinase HIPK2. DCAF7 is an evolutionarily conserved protein with a single WD40 repeat domain and has no catalytic activity. We have defined a DCAF7 binding motif of 12 amino acids in the N-terminal domain of class 1 DYRKs that is functionally conserved in DYRK1 orthologs from *Xenopus*, *Danio rerio* and the slime mold *Dictyostelium discoideum.* A similar sequence was essential for DCAF7 binding to HIPK2, whereas the closely related HIPK1 family member did not bind DCAF7. Immunoprecipitation and pulldown experiments identified DCAF7 as an adaptor for the association of the adenovirus E1A protein with DYRK1A and HIPK2. Furthermore, DCAF7 was required for the hyperphosphorylation of E1A in DYRK1A or HIPK2 overexpressing cells. Our results characterize DCAF7 as a substrate recruiting subunit of DYRK1A and HIPK2 and suggest that it is required for the negative effect of DYRK1A on E1A-induced oncogenic transformation.

The specific and regulated phosphorylation of target proteins by protein kinases is critical for all cellular functions including proliferation and differentiation. Mechanisms that ensure the spatiotemporal control of kinase signalling include the association of kinases with regulatory or targeting subunits and the formation of complexes with scaffolding and adaptor proteins^1^,^2^. Such regulatory proteins can allosterically modulate catalytic activity, localize the kinase to distinct subcellular compartments, or function as organizing platforms that recruit the kinase and the substrate to the same complex.

Dual-specificity tyrosine (Y)-phosphorylation regulated kinase 1A (DYRK1A) is a ubiquitously expressed protein kinase with pleiotropic functions in cellular regulation, including cell cycle control, cell survival, neural proliferation and differentiation^3^,^4^. Altered expression levels of DYRK1A, either due to heterozygous loss-of-function mutations or the presence of a third allele in Down syndrome, result in severe neurological alterations and impaired brain development in humans and mice^5^,^6^. DYRK1A belongs to a conserved family of protein kinases, which is comprised of 5 mammalian members that are further subdivided into either class 1 (including DYRK1A and DYRK1B) or class 2 DYRKs (DYRK2-4)^4^. DYRKs phosphorylate substrates on serine/threonine residues, but share the characteristic ability to autophosphorylate an activation loop tyrosine residue during translation^7^,^8^,^9^. In contrast to the regulatory tyrosine phosphorylation of the mitogen-activated protein kinases (MAPK), tyrosine phosphorylation in the DYRKs seems to be constitutive, and thus does not function as a control switch that regulates the activity of the mature kinase, e.g. in response to extracellular signals. The question arises as to how DYRK1A is controlled and what roles DYRK1A-interacting proteins may have in the regulation of its cellular function.

DCAF7 (also designated HAN11 or WDR68) is a WD40 domain protein that was discovered in a preparation of DYRK1A from rabbit skeletal muscle^10^. WD40 domains are characterized by several WD40 repeats, which are sequences of 44–60 amino acids in length that often harbour a defining tryptophan-aspartate (WD) dipeptide. These repeats form a circularized β*-*propeller structure that provide multiple binding surfaces for diverse protein–protein interactions and are found in numerous scaffold proteins^11^,^12^. *DCAF7* is the gene symbol assigned by the Human Gene Nomenclature Committee, meaning “DDB1 and CUL4 associated factor 7”. This name refers to the identification of the protein in DDB1 complexes and the deduced function as a substrate receptor in CLR4 E3 ubiquitin ligase complexes (CUL4–RBX1–DDB1)^13^. However, this role of DCAF7 has not yet been experimentally verified. DCAF7 has been repeatedly identified as a protein that co-purifies with DYRK1A^14^,^15^,^16^,^17^ and was also shown to bind DYRK1B^10^,^16^. The interaction with DCAF7 was narrowed down to the N-terminal region of DYRK1A^16^, which is partially conserved in class 1 DYRKs, but diverges in class 2 DYRKs. In accordance with this finding, DCAF7 does not bind to DYRK2, DYRK3 or DYRK4^16^. DCAF7 was also identified as a binding partner of HIPK2 (homeodomain interacting protein kinase 2)^18^, which is a neighbour of the DYRK family in the kinase dendrogram but shows no apparent sequence conservation with class 1 DYRKs except for the kinase domain.

Studies in genetically tractable organisms have yielded functional evidence on the role of DCAF7 and the interaction with class 1 DYRKs. In the zebrafish *Danio rerio*, DCAF7 (official gene symbol *wdr68*) was identified as a gene essential for craniofacial development^19^. Downregulation of *dyrk1b* induced similar defects in endoderm formation as reported for *wdr68* mutant animals, supporting a functional interaction of these proteins in zebrafish development^20^. The DCAF7 ortholog in *Drosophila*, CG14614 (official gene symbol *wap*, wings apart, also called *riq*, riquiqui), is essential for normal wing-vein patterning and development of the adult jump muscle^21^. CG14614 associates with the *Drosophila* DYRK1 ortholog, MNB (encoded by the minibrain gene, *mnb*), and both genes were shown to control normal wing and leg growth by modulating the Salvador-Wart-Hippo (SWH) pathway^22^.

These results from zebrafish and *Drosophila* provide evidence that the interaction of class 1 DYRKs with DCAF7 is functionally important in the modulation of pathways involved in differentiation and proliferation. Little is presently known about the function of DCAF7 in mammalian cells and the molecular mechanisms by which DCAF7 influences the function of DYRK1A. Interestingly, nuclear access of DCAF7 is required to maintain normal craniofacial development in zebrafish development^23^. Nevertheless, DCAF7 does not alter the distribution of DYRK1A between the nucleus and the cytoplasm^16^,^19^.

Mammalian DYRK1A and DCAF7 interact with human adenovirus type 5 (HAdV-5) early region 1A (E1A) protein^15^,^24^. E1A has been extensively characterized as an oncogene that is capable of immortalizing primary rodent cells and transforming them in cooperation with E1B or other oncogenes such as *ras*^25^. The interaction site with the DYRK1A/DCAF7 complex has been mapped to a conserved sequence in the C-terminal region of E1A^15^,^26^. Disruption of the DYRK1A/DCAF7-binding region increased the transforming activity of E1A in cooperation with *ras*, strongly suggesting that the interaction with DYRK1A attenuates the proliferative effect of E1A. E1A stimulates E2F-dependent transcription and S-phase entry by its interaction with the retinoblastoma (Rb) protein, whereas DYRK1A is a negative regulator of S-phase entry^3^,^27^,^28^. The role of DCAF7 in this process is unknown.

In the present study we have characterized the role of DCAF7 as an adaptor protein of class 1 DYRKs and HIPK2. We have defined the DCAF7 binding region in the N-terminal domains of DYRK1A and HIPK2 and show that the binding interface is functionally conserved even in a DYRK kinase from slime mold. Furthermore, we show that DCAF7 binds to E1A and acts as an essential adaptor protein that mediates the interaction of DYRK1A and HIPK2 with E1A.

## Results

### DCAF7 binds to DYRK1A, DYRK1B and HIPK2

**Figure 1:**
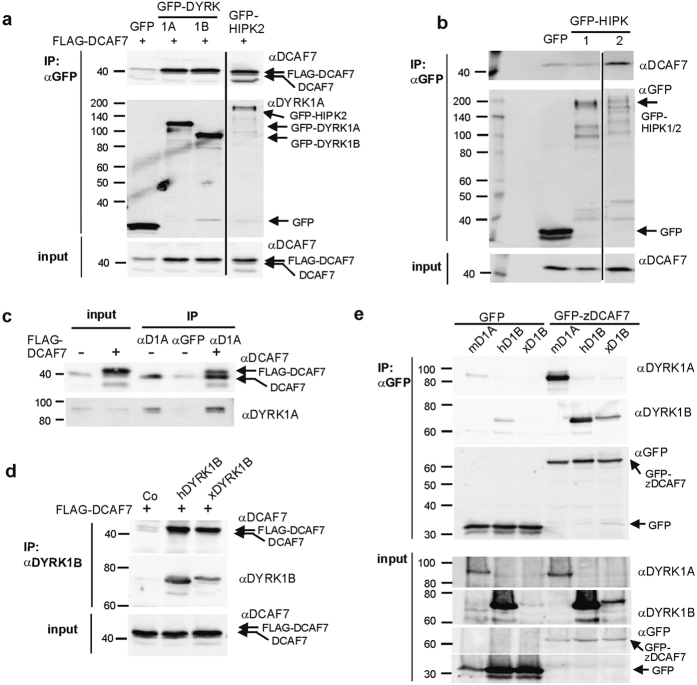
DCAF7 interacts with class 1 DYRKs and HIPK2. (**a**) Co-IP of human DCAF7 with rat DYRK1A, human DYRK1B and human HIPK2. - HeLa cells were transfected to co-express FLAG-hDCAF7 and the indicated GFP-fused protein kinases or GFP as a negative control. Total cell lysates were subjected to IP with GFP-trap beads and bound proteins were detected by immunoblotting with the antibodies directed against DCAF7 and GFP (αDCAF7 and αGFP). Aliquots of the whole cell lysates are shown as *input*. (**b**) HIPK1 does not bind DCAF7. – GFP, GFP-HIPK1 and GFP-HIPK2 were immunoprecipitated from transiently tranfected HeLa cells and analysed by Western blotting. (**c**) Co-IP of endogenous DYRK1A and DCAF7. – The lysate of untransfected HeLa cells was split before overnight IP with either goat anti DYRK1A (αD1A) or an unrelated goat antibody (αGFP). HeLa cells expressing FLAG-DCAF7 served as a positive control. (**d**) Co-IP of human DCAF7 with *Xenopus laevis* DYRK1B. – FLAG-hDCAF7 was co-expressed in HeLa cells with untagged *Xenopus* DYRK1B (xDYRK1B), human DYRK1B (hDYRK1B) as a positive control or empty vector as a negative control (Co). Binding of DCAF7 to immunoprecipitated hDYRK1B and xDYRK1B was detected by Western blot analysis. Note that the amounts of human and *Xenopus* DYRK1B cannot be directly compared because the epitope is only partially conserved in the *Xenopus* ortholog. (**e**) Co-IP of rat DYRK1A, human DYRK1B and *Xenopus* DYRK1B with zebrafish DCAF7. - GFP-tagged zebrafish DCAF7 was co-expressed in HeLa cells with murine FLAG-DYRK1A, hDYRK1B or xDYRK1B. GFP-trap was used to precipitate zDCAF7 and binding partners. The vertical lines indicate where irrelevant lanes were deleted from the final images.

GFP-tagged constructs of DYRK1A/B and HIPK2 were co-expressed with DCAF7 in HeLa cells to directly compare the interaction of these kinases with DCAF7 in co-immunoprecipitation (co-IP) experiments. Figure 1a shows that DCAF7 bound to HIPK2 as well as to the class 1 DYRKs. Interestingly, HIPK1 did not associate with DCAF7 (Fig. 1b), although HIPK1 and HIPK2 are closely related and share the same domain architecture^29^. Immunoprecipitation of endogenous DYRK1A from HeLa cells confirmed that DCAF7 interacts with DYRK1A at physiological expression levels (Fig. 1c).

Zebrafish DYRK1B has been reported to interact with the orthologous DCAF7 protein (also termed WDR68^20^), suggesting that the binding interface between DCAF7 and class 1 DYRKs is conserved in evolution. To test this, we determined whether the DCAF7 and class 1 DYRK orthologs from various species where able to interact with each other. We found that human DCAF7 was co-immunoprecipitated with *Xenopus* DYRK1B (Fig. 1d). Furthermore, mouse DYRK1A, human DYRK1B and *Xenopus* DYRK1B bind to GFP-DCAF7 from zebrafish (Fig. 1e). These results show that the structural determinants required for the interaction of DYRK1 with DCAF7 are conserved in vertebrates.

### Identification of the DCAF7 binding site in DYRK1A

**Figure 2:**
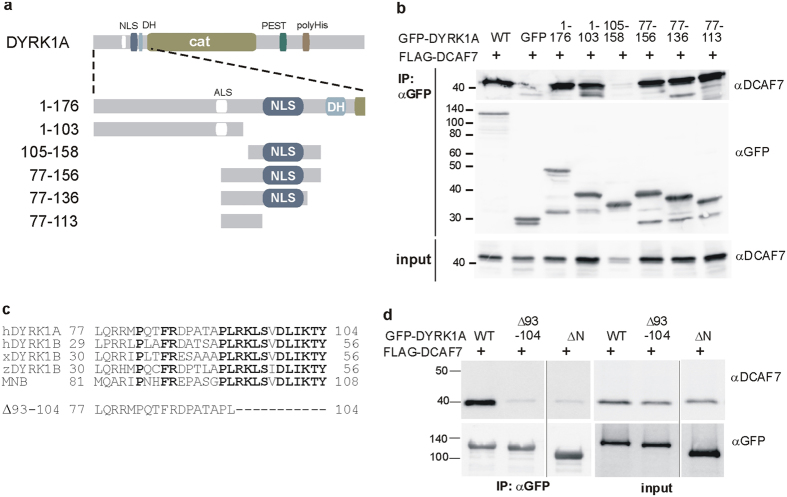
Mapping of the DCAF7-interacting sequence in DYRK1A. (**a**) Schematic representation of DYRK1A domain structure and deletion clones. - Deletion constructs tested for DCAF7 binding are illustrated in a close-up of the N-terminal domain. ALS, alternatively spliced segment of 9 amino acids that is absent in the short splicing variant; NLS, nuclear localization sequence; DH, DYRK homology box; cat, catalytic domain; PEST, proline-, glutamic acid-, serine-, threonine-rich region; polyHis, poly-histidine tract. (**b**) Co-IP of FLAG-DCAF7 with GFP-DYRK1A deletion constructs. - HeLa cells co-expressing FLAG-DCAF7 and the indicated GFP-DYRK1A deletion constructs or unfused GFP were used for GFP-IP. Binding of DCAF7 was analysed by immunoblotting. (**c**) Alignment of the minimal DCAF7 binding region in vertebrate class 1 DYRKs and the *Drosophila* ortholog MNB. - A deletion was introduced in the full length kinase to narrow down the binding region (Δ93–104). (**d**) FLAG-DCAF7 does not bind to GFP-DYRK1A-Δ93–104. – Binding of FLAG-DCAF7 to GFP-DYRK1A constructs was assayed by co-IP as in panel (**b**). GFP-DYRK1A-ΔN lacks the N-terminal domain (Δ1–134) and was used as a negative control.

The N-terminal domain of DYRK1A was shown to be essential for binding of DCAF7^16^. To narrow down the critical region, we created a panel of DYRK1A deletion constructs (Fig. 2a) and examined their capacity to bind DCAF7 (Fig. 2b). These experiments identified a region of 27 amino acids located N-terminal to the nuclear localization signal that was essential for the formation of the DCAF7/DYRK1A complex. Within this region, a motif comprising residues 92–104 was conserved among all class 1 DYRKs that are known to interact with DCAF7 (Fig. 2c). A deletion mutant of DYRK1A lacking amino acids 93–104 did not bind to DCAF7, confirming that this sequence was necessary for interaction (Fig. 2d). Regardless of the close vicinity of the nuclear localization signal, the deletion mutant did not differ from the wild type protein in its near exclusive nuclear localization (supplementary Fig. S1). Further attempts to define the structural determinants of the DCAF7-binding interface in DYRK1A by alanine scanning revealed that no single residue in this region was essential for binding (supplementary Fig. S2).

### The DCAF7 binding site is conserved in DYRK1 from slime mould

**Figure 3:**
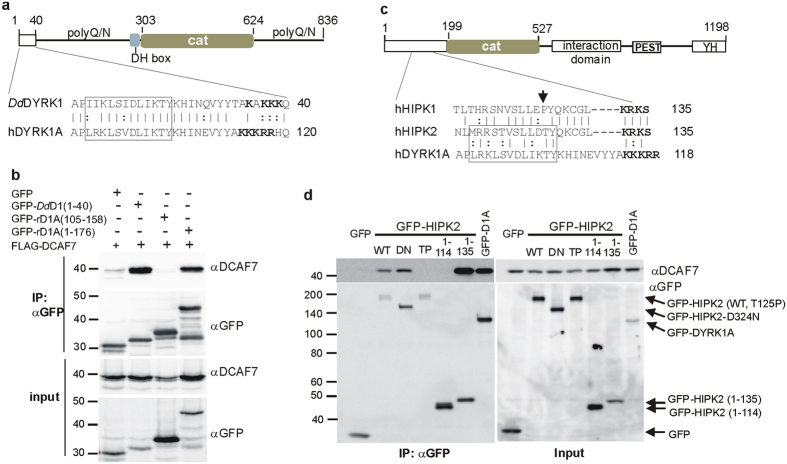
Mapping of DCAF7-binding sequences in *Dictyostelium* DYRK1 and human HIPK2. (**a**) Structure of the DYRK1 ortholog in *Dictyostelium discoideum* (*Dd*DYRK1A, Uniprot Q76NV1). - The sequence encoded by the first exon shows similarity with the DCAF7 binding region of human DYRK1A (boxed). (**b**) Co-IP of human DCAF7 with *Dd*DYRK1A. - HeLa cells expressing a GFP fusion protein of the N-terminal region DdDYRK1A (amino acids 1–40) and human FLAG-DCAF7 were used for anti GFP IP. DYRK1A deletion constructs served as negative and positive controls. (**c**) Structure of human HIPK2 (Uniprot Q9H2X6). - The N-terminal domain of HIPK2 harbours a motif weakly similar to the DCAF7 binding region of DYRK1A. The corresponding sequence in HIPK1 is shown for comparison. The arrowhead points to a proline in the HIPK1 sequence that is not found in HIPK2. (**d**) Co-IP of DCAF7 with GFP-HIPK2 deletion constructs and point mutants. - GFP, GFP-HIPK1 and GFP-HIPK2 were immunoprecipitated from transiently tranfected HeLa cells and analysed by Western blotting for the binding of endogenous DCAF7. GFP and GFP-DYRK1A served as negative and positive controls. HIPK2-D324N is a catalytically inactive point mutant of HIPK2. DH, DYRK homology box; cat, catalytic domain; poly Q/N, polyglutamine/polyasparagine tracts; PEST, region rich in proline, glutamic acid, serine and threonine residues; YH, tyrosine/histidine-rich region. Putative nuclear localization signals are highlighted in bold print.

Database searches revealed that a DYRK1 orthologous kinase in the slime mould *Dictyostelium discoideum* (*Dd*DYRK1A) harbors a sequence motif very similar to the DCAF7-binding region of DYRK1A (Fig. 3a). To test whether this sequence motif was a functional DCAF7 binding site, we constructed a GFP fusion protein comprising the N-terminal region of *Dd*DYRK1A (amino acids 1–40). In co-IP experiments, human DCAF7 bound as well to the *Dictyostelium* sequence as to the corresponding region from human DYRK1A (Fig. 3b). This result shows that the binding interface of the DYRK1/DCAF7 complex is extremely well conserved, supporting the hypothesis that the association with DCAF7 plays a fundamental role for the function of class 1 DYRKs.

### Mapping of the DCAF7 binding site in HIPK2

Deletion of the N-terminal domain of HIPK2 had been shown to abolish DCAF7 binding^18^. However, HIPK2 shares no obvious sequence similarity with class 1 DYRKs outside the catalytic domain. Visual analysis identified a short segment within the N-terminal region as the best potential match with amino acids 93–104 from DYRK1A (Fig. 3c). This motif is conserved in all vertebrate HIPK2 sequences, while the corresponding sequence in HIPK1 differs in several positions. Co-IP experiments showed that DCAF7 bound to a GFP fusion protein containing the first 135 amino acids of HIPK2, but not to a shorter construct comprising amino acids 1–114 (Fig. 3d). This result localizes the DCAF7 binding site of HIPK2 to a segment of 21 amino acids in the N-terminal region of HIPK2. Considering the difference in DCAF7 binding between HIPK1 and HIPK2, we speculated that the presence of a proline in the corresponding sequence of HIPK1 might disrupt the binding interface (Fig. 3c). Indeed, the substitution of Thr125 by proline abolished DCAF7 binding to HIPK2 (Fig. 3d). As with DYRK1A, DCAF7 binding did not depend on the catalytic activity of HIPK2, since the interaction was maintained by the kinase deficient HIPK2_D324N_ point mutant (Fig. 3d). The higher electrophoretic mobility of this mutant results from its reduced autophosphorylation^30^.

### DCAF7 is required for efficient binding of E1A to DYRK1A, DYRK1B and HIPK2

**Figure 4:**
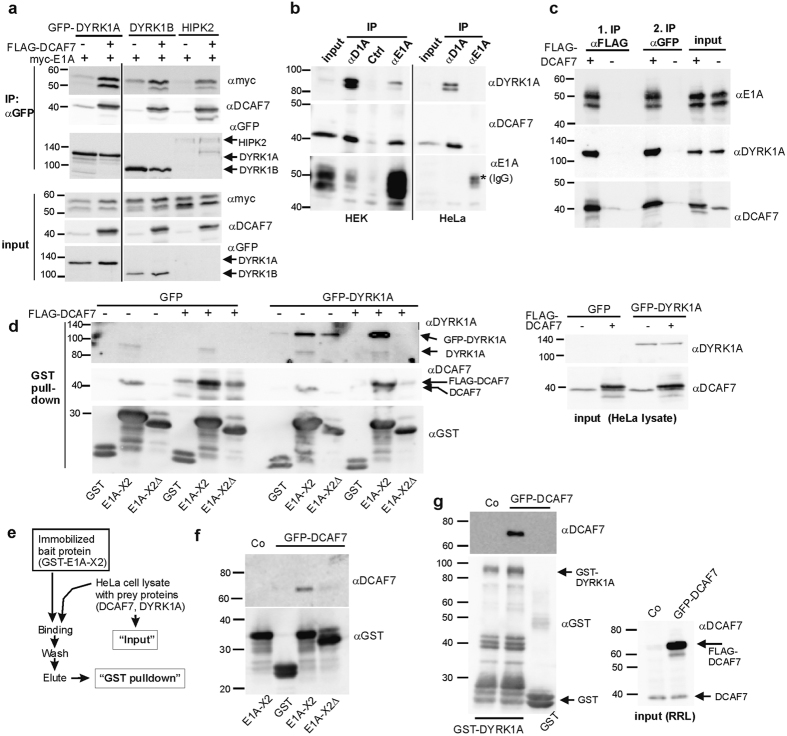
DCAF7 mediates binding of E1A to DYRK1A, DYRK1B and HIPK2. (**a**) Co-IP of myc-E1A with DYRK1A, DYRK1B, HIPK2 and DCAF7. - HeLa cells were transfected to co-express myc-E1A (289 amino acid form) GFP-DYRK1A, DYRK1B or HIPK2 and either FLAG-DCAF7 or a control vector. The vertical line indicates where irrelevant lanes were deleted from the final image. Note that GFP-HIPK2 is difficult to reveal on the blots due to its large size and could not be detected in the cell lysates (input). (**b**) Co-IP of endogenous DYRK1A/DCAF7 with E1A. - The lysate of untransfected HEK293 cells or HeLa cells was subjected to IP with either goat anti DYRK1A (αD1A) or mouse anti-E1A (αE1A). An unrelated goat antibody (Ctrl) was used as a negative control for the IP with αD1A. HeLa cells (which lack E1A) were used as background control for the αE1A IP. The heavy chain of the immunoprecipitating E1A antibody is marked by an asterisk (IgG). (**c**) DYRK1A, DCAF7 and E1A are components of a common complex. - HEK293-(GFP-DYRK1A-tetOn) cells were transfected with a FLAG-DCAF7 expression vector or empty control plasmid and induced with doxycyclin to express GFP-DYRK1A. Lysates were subjected to sequential IP with anti FLAG and anti GFP. The two E1A bands may correspond to the major protein forms of 289 and 243 amino acids that are expressed in HEK293 cells. (**d**) GST pulldown assay. - HeLa cells were transfected to express GFP or GFP-DYRK1A either with FLAG-DCAF7 or alone. Aliquots of cell lysates were used for pulldown assays with agarose-bound GST or a GST-tagged construct of the exon2-encoded portion of E1A (E1A-X2) as bait. A deletion mutant (X2Δ, deletion of amino acids 255–270) that lacks the DYRK1A/DCAF7 binding region of E1A served as negative control. (**e**) Outline of the pulldown assay (**f**,**g**) *In vitro* interaction assays. – GFP-DCAF7 was *in vitro*-translated in rabbit reticulocyte lysate (RRL) and used as a prey for GST-pulldown assays with immobilized GST fusion proteins as indicated below the bottom panel. In parallel control samples (Co), *in vitro* transcription was driven by the empty vector.

The E1A protein from HAdV-5 interacts with DYRK1A and DCAF7^24^,^15^,^26^. To investigate the role of DCAF7 as an adaptor protein in this complex, we conducted co-IP experiments in HeLa cells co-expressing myc-tagged E1A and GFP-tagged constructs of DYRK1A, DYRK1B and HIPK2 with either FLAG-DCAF7 or a control plasmid (Fig. 4a). All three kinases formed complexes with E1A and DCAF7, while only very weak binding of E1A was observed without DCAF7 overexpression. As the association of E1A with HIPK2 has not been previously reported, we used the HEK293 cell line to confirm that endogenous E1A was also associated with HIPK2/DCAF7 (supplementary Fig. S3). The HEK293 clone was originally immortalized by transformation with fragmented HAdV-5 DNA and constitutively expresses the two major E1A splice variants of 243 (E1A 12S) and 289 (E1A 13S) amino acids. Next, we used untransfected HEK293 cells to analyze the interaction of DYRK1A, DCAF7 and E1A under normal expression levels of all proteins. This experiment showed that a small amount of the endogenous E1A associates with DYRK1A/DCAF7 complexes (Fig. 4b). Furthermore, both DYRK1A and DCAF7 were found in anti-E1A IP from HEK293, but not in otherwise identical experiments performed in HeLa cells, which do not express E1A (Fig. 4b).

To prove that DCAF7 can simultaneously bind DYRK1A and E1A, we took advantage of a HEK293 derivative line that stably overexpresses GFP–DYRK1A under the control of a doxycyclin-regulatable promoter. After transient expression of FLAG-DCAF7, cell lysates were subjected to successive IP with anti-FLAG and anti-GFP antibodies. Following anti FLAG IP, bound proteins were eluted from the affinity gel with an excess of FLAG peptide, and the eluates were incubated with GFP trap paramagnetic beads to capture FLAG-DCAF7/GFP-DYRK1A complexes. Both forms of the E1A proteins were detected in the final immunoprecipitate (Fig. 4c), indicating that DYRK1A, DCAF7 and E1A are components of the same protein complex in HEK293 cells.

To further characterize this complex, we conducted a series of GST-pulldown assays. The region of E1A responsible for interaction with DCAF7/DYRK1A has been mapped to a short sequence including residues 250–277 encoded by exon 2 (X2)^15^,^26^. Therefore, we used recombinant GST-fused E1A-X2 as bait to pull down DYRK1A and DCAF7 from HeLa cell lysates (Fig. 4e). GST and a mutant of E1A with a deletion of the DYRK1A-binding region served as background controls. Non-specific binding was detected in some control lanes but was much weaker than specific binding of DCAF7 to E1A-X2 (Fig. 4d). Overexpression of FLAG-DCAF7 markedly increased the binding of GFP-DYRK1A to E1A-X2, while binding of endogenous DYRK1A was unaffected by FLAG-DCAF7. This result is consistent with the assumption that endogenous DYRK1A exists already in a stoichiometric complex with endogenous DCAF7, whereas binding of overexpressed GFP-DYRK1A depends on the presence of overexpressed FLAG-DCAF7. In contrast, binding of FLAG-DCAF7 to E1A was not affected by the presence or absence of GFP-DYRK1A. Similarly, binding of E1A-X2 to immobilized GST-DYRK1A depended on the presence of DCAF7, whereas the association of DCAF7 with DYRK1A was not affected by the presence of E1A-X2 (supplementary Fig. S4).

The pulldown of DCAF7/DYRK1A from HeLa lysates by GST-E1A does not prove direct binding of these proteins, since the complex may include one or more additional cellular proteins. Therefore, we produced GFP-DCAF7 by *in vitro*-translation for use as a prey in GST pulldown experiments. As shown in Fig. 4f,g, GFP-DCAF7 bound to bacterially expressed GST-E1A-X2 and GST-DYRK1A, but not to GST or GST-E1A-X2_Δ250–277_). This result strongly suggests that DCAF7 directly binds to E1A and DYRK1A, although we cannot fully rule out the possibility that other proteins present in the rabbit reticulocyte lysate participate in the complex.

**Figure 5:**
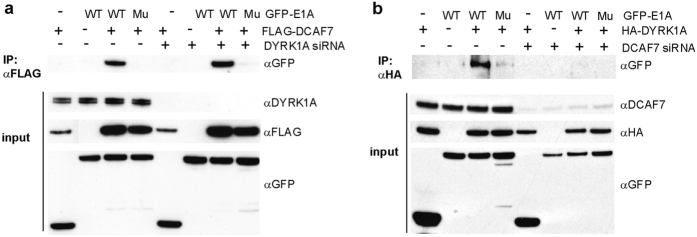
DCAF7 is an essential adaptor protein for the association of E1A with DYRK1A *in vivo*. Human HT1080 cells were treated with control siRNA, siRNA specific to DYRK1A (**a**) or siRNA specific to DCAF7 (**b**). Cells were subsequently co-transfected with the blank vector, GFP-E1A (WT, 289 amino acid form) or the E1A point mutant R262/263E (Mu) and vectors expressing FLAG-DCAF7 (**a**) or HA-DYRK1A (**b**). The E1A-R262/263E mutant does not interact with DYRK1A or DCAF7^26^ and served as a specificity control. Lysates were immunoprecipitated using anti-FLAG antibodies (**a**) or anti-HA antibodies (**b**) and immunoblotted using anti-GFP antibodies to detect the presence of co-precipitating E1A.

We performed knockdown experiments to further determine the roles of DYRK1A and DCAF7 in forming a complex with E1A. Co-IP of E1A with FLAG-DCAF7 was not affected by knockdown of endogenous DYRK1A (Fig. 5a), whereas knockdown of DCAF7 eliminated binding of E1A to HA-DYRK1A (Fig. 5b). These results conclusively identify DCAF7 as an adaptor protein that is necessary for the association of E1A with DYRK1A.

### Structural basis of the E1A-DYRK1A and E1A-HIPK2 binding

**Figure 6:**
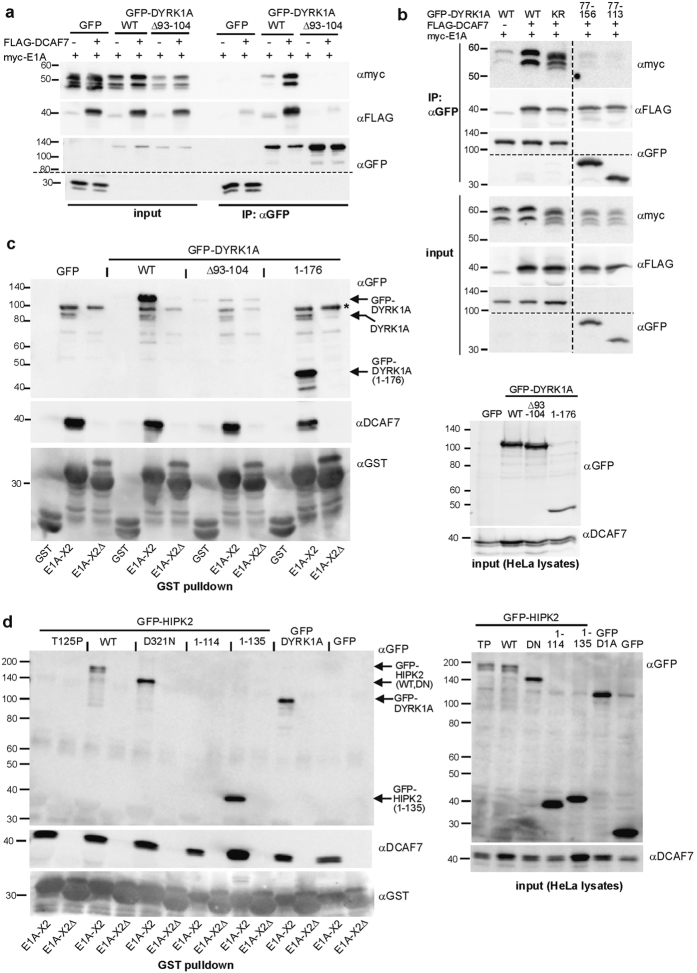
Structural basis for E1A binding to DYRK1A and HIPK2. (**a**,**b**) Co-IP of myc-E1A wild type DYRK1A, deletion mutants of DYRK1A and a kinase-negative point mutant of DYRK1A (K188R). - Lysates of HeLa cells expressing myc-E1A, FLAG-DCAF7 and the indicated GFP-DYRK1A constructs were subjected to anti GFP IP. The dashed lines indicate where irrelevant regions of the blots were deleted from the final image. (**c**,**d**) GST pulldown assays. - HeLa cells were transfected to co-express FLAG-DCAF7 with GFP-DYRK1A constructs or GFP-HIPK2 constructs as indicated. Cell lysates were subjected to GST-pulldown assay with immobilized GST or GST-E1A-X2 and bound proteins were analysed by immunoblotting. GST-E1A-X2Δ lacks the DYRK1A/DCAF7 binding region of E1A and served as negative control. To reveal the GFP-DYRK1A_1–176_ construct, a polyclonal goat antibody directed against an N-terminal epitope was used in panel (**c**). This antibody crossreacts with an unidentified band (marked by an asterisk) that is not detected by the monoclonal DYRK1A antibody used in Fig. 4d. Endogenous DYRK1A is detected as a double band at ~90 kDa.

We analyzed the binding of E1A to DYRK1A deletion constructs to further define the structural requirements for this interaction. As expected, GFP-DYRK1A_Δ93–104_, which is unable to bind DCAF7, did not bind E1A (Fig. 6a). This confirmed that the interaction with DCAF7 is necessary for DYRK1A to bind E1A. Next we tested truncated DYRK1A constructs that contained the DCAF7 interaction site (Fig. 6b). These deletion constructs showed minimal or no binding of E1A (Fig. 6b, suppl. Fig. S5), although DCAF7 was efficiently precipitated. Thus, binding of DCAF7 to DYRK1A was necessary, but not sufficient, to establish an interaction with E1A that was stable enough to be detected by co-IP. Catalytic activity was not required for the assembly of the complex, since the kinase deficient DYRK1A_K188R_ mutant showed strong binding to E1A. Interestingly, co-expression of wild type DYRK1A, but not the catalytically inactive mutant, resulted in a reduced electrophoretic mobility of the E1A bands (Fig. 6b), suggesting that DYRK1A phosphorylates E1A.

We performed pulldown experiments to explore the formation of the DYRK1A/DCAF7/E1A complex under *in vitro* conditions (Fig. 6c). GFP-DYRK1A_Δ93–104_ was not pulled down by recombinant E1A-X2, once more providing evidence that DCAF7 acts as an essential adaptor for this interaction. In contrast to the co-IP results, the interaction of GFP-DYRK1A_1–176_ with E1A-X2 was clearly detectable under the conditions of this assay. Similar results were obtained with other deletion constructs including GFP-DYRK1A_77–113_, which contains essentially little more than the DCAF7 binding motif of DYRK1A (supplementary Fig. S6). This discrepancy between the co-IP result and the pulldown assay was consistently observed in replicate experiments. Of note, the complex of E1A with DCAF7/DYRK1A forms *in vitro* with an excess of immobilized E1A in the case of the pulldown experiment. In contrast, the co-IP identifies complexes that have formed *in vivo* in the presence of other potentially competing binding partners. It appears plausible that regions other than the DCAF7 binding site of DYRK1A support the formation of the DYRK1A/DCAF7/E1A under physiological conditions, but are not required under *in vitro* conditions.

We also performed pulldown assays to further characterize the role of DCAF7 in the E1A/HIPK2 complex (Fig. 6d). As above, we used the GST-E1A-X2 fusion protein as bait and an E1A mutant deficient in DCAF7 binding (X2Δ) as background control. Binding of GFP-HIPK2 to E1A-X2 depended on the presence of the DCAF7-interaction motif in E1A and was abrogated by the T125P mutation in HIPK2. Furthermore, HIPK2_1–135_ was pulled down by E1A-X2, but HIPK2_1–114_ was not, again demonstrating a requirement for the DCAF7 binding motif. Thus, DCAF7 functions as an adaptor in protein complexes of E1A with HIPK2 as well as DYRK1A.

### Phosphorylation of E1A

**Figure 7:**
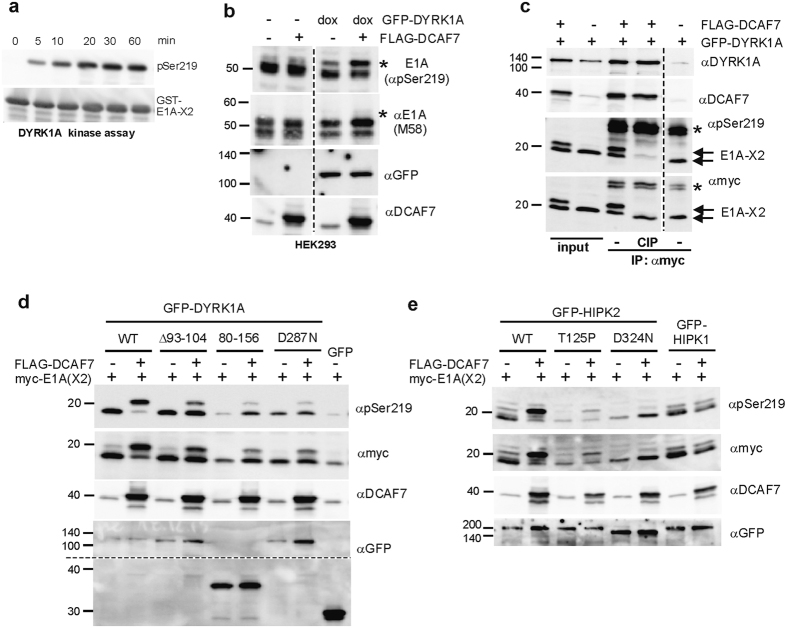
Phosphorylation of E1A by DYRK1A and HIPK2. (**a**) *In vitro* kinase assay. - Bacterially expressed GST-E1A-X2 was incubated with recombinant DYRK1A at 30 °C in the presence of 1 mM ATP. Aliquot of the reaction were taken at variable times and phosphorylation of E1A was detected by Western blot analysis with a phosphospecific antibody directed against pSer219. (**b**) Phosphorylation of E1A in HEK293 cells. - HEK293-(GFP-DYRK1A-tetOn) cells were transfected with a FLAG-DCAF7 expression vector or empty control plasmid and either induced with doxycyclin (dox) to express GFP-DYRK1A or not induced. Two days after transfection, total cellular lysates were analyzed for Ser219 phosphorylation. Detection of E1A by the monoclonal antibody M58 is independent of the phosphorylation state. The asterisks mark an upshifted band in DYRK1A overexpressing samples. (**c**–**e**) Phosphorylation of E1A exon2 by DYRK1A and HIPK2. - HeLa cells were co-transfected with expression plasmids for myc-E1A-X2, FLAG-DCAF7 and DYRK1A, HIPK1, HIPK2 or mutant kinase constructs as indicated. In **c**, myc-E1A-X2 was immunoprecipitated and either dephosphorylated by calf intestinal phosphatase (CIP) or not treated before SDS-PAGE. The asterisks mark the light chain bands of the immunoprecipitating antibody. In (**d**,**e**), total cellular lysates were analysed for phosphorylation of E1A-X2 2 days after transfection.

We had previously identified a peptide mimicking the sequence around Ser219 in E1A as one of the best *in vitro* substrates of DYRK1A among 720 peptides (suppl. data in ref. 31). Moreover, this site in E1A matches the optimal DYRK1A target sequence (RPXSP)^32^ and is essential for the interaction of FOXK1 and FOXK2 with E1A^15^. Kinase assays with recombinant GST-E1A-X2 confirmed that DYRK1A efficiently phosphorylates Ser219 *in vitro*, as detected by a phosphospecific antibody (Fig. 7a). However, overexpression of GFP-DYRK1A did not increase the phosphorylation of Ser219 in HEK293 cells, which was already high in the absence of exogenous DYRK1A (Fig. 7b). Nevertheless, overexpression of DYRK1A and DCAF7 induced the appearance of an E1A form with reduced electrophoretic mobility. This result suggests that DYRK1A phosphorylates E1A on sites other than Ser219.

To narrow down the region of this phosphorylation event, we used an E1A deletion construct comprising only the region encoded by exon 2 (myc-E1A-X2). Overexpression of both DYRK1A and DCAF7, but not DYRK1A alone, resulted in a marked mobility shift of E1A-X2 (Fig. 7c, input lanes). The change in mobility was not caused by the phosphorylation of Ser219, since both the faster and slower migrating bands are detected by the pSer219 specific antibody. Treatment with phosphatase eliminated the upper band, indicating that the altered electrophoretic mobility results from phosphorylation on other site(s) (Fig. 7c).

The same upshift of myc-E1A-X2 was induced by overexpression of HIPK2 and DCAF7 (suppl. Fig. S7). Furthermore, this phosphorylation of E1A by DYRK1A and HIPK2 was strictly dependent on the presence of the DCAF7 binding sequence in E1A (amino acids 255–270). Analysis of mutant DYRK1A constructs showed that the maximal effect on E1A electrophoretic mobility depended both on the catalytic activity of DYRK1A and on the capacity to bind DCAF7 (Fig. 7d). A weak effect of DCAF7 overexpression was observed in the presence of catalytically inactive DYRK1A constructs, which may be due to recruitment of endogenous kinases (DYRK1A, DYRK1B or HIPK2) to E1A. As observed for DYRK1A, the phosphorylation of E1A by HIPK2 was dependent on kinase activity and on DCAF7 binding (Fig. 7e). The requirement for DCAF7 was further supported by the fact that HIPK1 did not induce the mobility shift of E1A.

### Subcellular localization of the E1A/DCAF7/DYRK1A complexes

**Figure 8:**
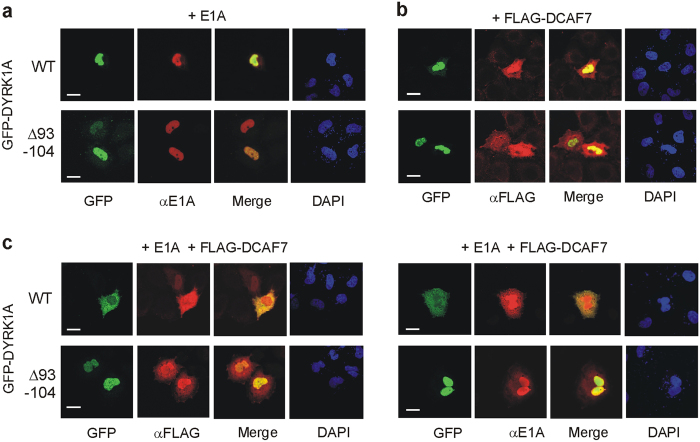
Subcellular localization of the E1A/DCAF7/DYRK1A complexes. HeLa cells transiently transfected to co-express either wild type GFP-DYRK1A (WT) or GFP-DYRK1A-Δ93–104 together with E1A (243 amino acid form) and/or FLAG-DCAF7. Proteins were detected by autofluorescence (GFP) or immunofluorescence (E1A, FLAG-DCAF7). Nuclei were stained with DAPI. Scale bar, 100 μm.

Immunofluorescence experiments were performed to address the question whether the interaction of E1A, DCAF7 and DYRK1A altered the intracellular localization of the individual proteins. Wild type GFP-DYRK1A and GFP-DYRK1A_Δ93–104_ were co-localized in the nucleus with E1A, and the overexpression of E1A had no apparent effect on either DYRK1A construct (Fig. 8a, compare suppl. Fig. S1). In contrast, FLAG-DCAF7 was only partially co-localized with DYRK1A in the nucleus and was also detectable in the cytoplasm (Fig. 8b). Strikingly, co-expression of both E1A and FLAG-DCAF7 resulted in an extensive relocalization to the cytoplasm of both DYRK1A and E1A (Fig. 8c). This effect was not observed with DYRK1A_Δ93–104_, providing further evidence that the DCAF7 binding site in the N-terminal domain of DYRK1A is able to direct the formation of complexes with E1A and DYRK1A in living cells.

## Discussion

In this study we have defined the DCAF7 binding region in the N-terminal domains of class 1 DYRKs and HIPK2. In addition, we show that the interaction with DCAF7 is highly conserved in class 1 DYRKs throughout evolution, from mammals, amphibians, fish and even in unicellular organisms like *Dictyostelium discoideum*. Furthermore, the present results identify DCAF7 as an adaptor protein that is necessary to establish complexes between the adenoviral E1A protein and the DYRK1A or HIPK2 kinases.

### The interaction between DCAF7 and class 1 DYRKs is conserved in evolution

**Figure 9:**
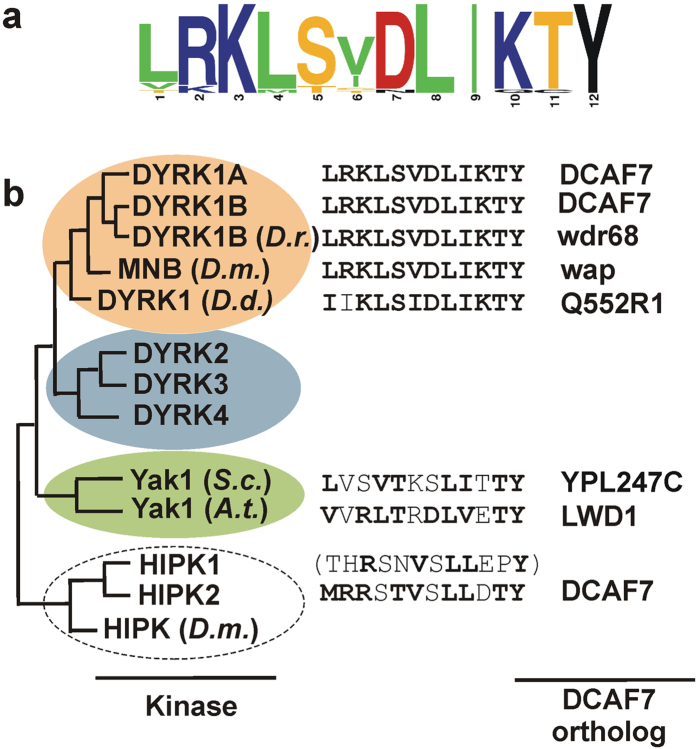
Evolutionary conservation of the DYRK/DCAF7 interaction. (**a**) Consensus sequence of the DCAF7 binding motif in class 1 DYRKs. - The sequence logo was created from an alignment^9^ of 19 representative sequences of class 1 DYRKs in the animal kingdom, including sponges, jellyfish, sea urchin, insects and different worms using the WebLogo application^52^. (**b**) Phylogenetic relationship of the DYRKs and HIPKs that interact with DCAF7. – Conservation of the DCAF7 binding sequence is illustrated for kinases that are known to bind DCAF7 or orthologous proteins. Mammalian DYRK2-4 and HIPK1 do not bind DCAF7. It is unknown whether *Drosophila* HIPK binds to the *wap* protein, but the DCAF7 binding site from HIPK2 is not conserved in invertebrates. Kinase branches from top to bottom: class 1 DYRKs, class 2 DYRKs, YAK branch of DYRKs, HIPKs. Non-mammalian kinases are from *Danio rerio* (*D.r.*)*, Drosophila melanogaster* (*D.m.*)*, Dictyostelium discoideum* (*D.d.*)*, Saccharomyces cerevisiae* (*S.c.*), and *Arabidopsis thaliana* (*A.t.*).

Our results localize the DCAF7 binding site in DYRK1A to a sequence of 27 amino acids (77–103) and identify a core motif of 12 amino acids (93–104) that is necessary for this interaction (Fig. 9a). Not only is this sequence highly conserved in evolution, but the DYRK1 ortholog from the slime mold *Dictyostelium discoideum* was able to bind to human DCAF7 as efficiently as mammalian DYRK1A. This result implies that the binding interface between class 1 DYRKs and DCAF7 orthologs is functionally conserved throughout evolution. The interaction with DCAF7 is thus a fundamental property of class 1 DYRKs, as further evidenced by the fact that the DCAF7 binding motif is the only conserved sequence in *Dd*DYRK1 outside the catalytic domain.

DCAF7 is highly conserved in eukaryotic organisms, and its orthologs interact with members of the DYRK family in zebrafish (WDR68 and DYRK1B)^19^, *Drosophila* (wings apart (*wap*) and minibrain (*mnb*))^22^ and *Arabidopsis thaliana* (LWD1 and *At*Yak1)^33^. Global interaction screens have also identified the yeast ortholog of DCAF7 (YPL247C) as a binding partner of YAK1^34^,^35^, which is the only member of the DYRK family in baker’s yeast. It should be noted that *At*YAK1 and YAK1 do not belong to class 1 DYRKs, but are members of a DYRK branch that is absent from the animal kingdom^4^ (Fig. 9b). Thus, the interaction with DCAF7 appears to be an ancestral feature of the DYRK family that was lost in class 2 DYRKs.

The remarkable conservation of the DCAF7/DYRK interaction is highlighted by results showing that human DCAF7 can partially complement a defect in flower pigmentation in petunia an11 mutants^36^ and that the *Drosophila* wap protein can rescue the defects in craniofacial development of zebrafish caused by WDR68 mutation^19^. Taking into consideration that DCAF7 was repeatedly co-purified with DYRK1A from various sources^14^,^15^,^16^,^17^,^37^, we hypothesize that DCAF7 functions as an important subunit of DYRK1A and other class 1 DYRKs. RACK1 (Receptor for Activated C Kinase 1) is a WD repeat scaffold protein that selectively interacts with PKC in the activated status^38^. In contrast, DCAF7 binds to DYRK1A and HIPK2 independently of kinase activity, although the serine residue (Ser97) within the DCAF7 binding motif is a well characterized autophosphorylation site of DYRK1A^39^. In this way, DCAF7 appears to function as a hub for protein-protein interactions, like the WD repeat domain of LRRK2 (leucine-rich repeat kinase 2)^40^. In accordance with this idea, DCAF7 coordinates signalling complexes by acting as an adaptor for proteins such as the cytoskeletal regulator mDia1^14^ and mitogen-activated protein kinase kinase kinase 1 (MEKK1)^18^. DCAF7 does not seem to act as an anchoring protein for its interacting kinases, because previous studies showed that DCAF7 is recruited to the nucleus by overexpressed DYRK1A or HIPK2^16^,^18^. Unexpectedly, our results show that the simultaneous overexpression of E1A and DCAF7 induced a massive redistribution of DYRK1A and E1A from the nucleus to the cytoplasm (Fig. 8c). The bipartite nuclear localisation sequences (NLS) of DYRK1A and E1A are located in close vicinity of the DCAF7 binding site (DYRK1A, Fig. 2a) or even overlap (E1A, ref. 41). It appears possible that steric hindrance by the interacting proteins in the DYRK1A/DCAF7/E1A complex shields the nuclear localisation sequences in DYRK1A and in E1A from interacting with the importin complex. The functional consequences of this redirection of DYRK1A and E1A to the cytoplasm remain to be elucidated.

### DCAF7 is an adaptor protein for the adenoviral E1A protein

**Figure 10:**
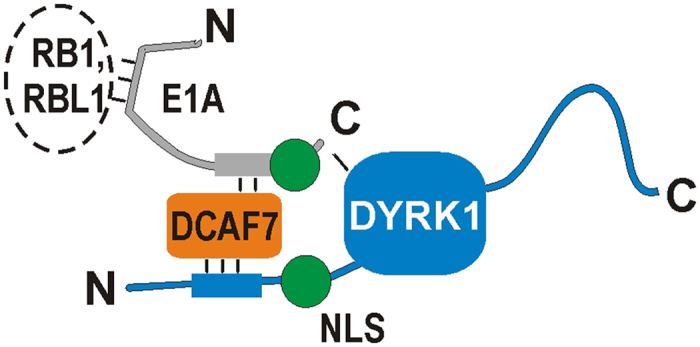
Proposed model of the DYRK1A-DCAF7-E1A complex. Multiple binding sites allow for the simultaneous interaction of DCAF7 with E1A and DYRK1. E1A is an intrinsically disordered protein that is known to interact with many cellular proteins including the pocket proteins (RB1, RBL1). NLS, nuclear localization signal.

Our present results characterize DCAF7 as an adaptor protein that mediates the association of DYRK1A with E1A (Fig. 10a). DCAF7 is a single-domain WD repeat protein, and computer-aided structural analysis strongly suggests that it forms a classical seven-bladed β-propeller fold that encompasses the whole polypeptide chain^42^. This structure serves as a rigid scaffold that allows WD domain proteins to interact with diverse proteins or peptides using multiple surfaces^11^. Our co-IP and pulldown experiments indicate that DCAF7 binds to E1A independently of the presence or absence of DYRK1A. Conversely, the interaction of DYRK1A with E1A depends on the presence of DCAF7 and the DCAF7 binding motif in DYRK1A. The capacity to simultaneously bind different proteins at independent sites is critical for the scaffolding function of β-propeller proteins^11^ and has been demonstrated in atomic detail for the WD protein WDR5^43^. Interestingly, the previous identification of YAK1 as an interaction partner of E1A in a yeast two hybrid screen implies that the endogenous DCAF7 ortholog of yeast (YPL247C) functioned as an adaptor between bait and prey^24^. This further underscores the extraordinary evolutionary conservation of the DCAF7-DYRK interaction.

Interactome studies in HEK293 cells have identified all major E1A binding proteins (i.e. RB1, RBL1, RBL2, EP300, EP400, CREBBP, CTBP2, FOXK1)^44^ as interaction partners of DYRK1A and DYRK1B^17^. This result suggests that DYRK1A/B modulates the function of E1A in adenovirus-infected cells. Indeed, the interaction of E1A with DCAF7/DYRK1A has a suppressive effect on the proliferation and transformation of adenovirus-infected cells^15^, which correlates with the function of DYRK1A as a negative regulator of the cell cycle^3^,^28^,^45^. DYRK1A also catalyzes the phosphorylation of E1A at Ser219 *in vitro*, which is prerequisite for the interaction of E1A with the FOXK1/2 transcription factors that inhibit oncogenic cell transformation^15^. FOXK1/K2 contain canonical forkhead associated domains, which function as phospho-specific protein–protein interaction motifs^46^. Thus, the interaction and subsequent phosphorylation of E1A by DYRK1A may facilitate the interaction of E1A with FOXK1/2. Despite serving as an excellent substrate *in vivo*, we failed to detect a significant effect of DYRK1A overexpression on pSer219 in cell assays. However, DYRK1A clearly phosphorylated E1A in a DCAF7-dependent manner on other sites, as evidenced by the appearance of a slower migrating E1A form in *in vivo* experiments (Fig. 7). Thus, E1A appears to hijack DCAF7 as an adaptor protein to facilitate its phosphorylation by DYRK1A *via* a mechanism of ‘enforced proximity’ between kinase and substrate. It remains to be shown whether DCAF7 also functions as a substrate recruiting subunit of class 1 DYRKs to phosphorylate non-viral target proteins under physiological conditions.

### Interaction of HIPK2 with DCAF7

Ritterhoff *et al.*^18^ have characterized DCAF7 as a scaffold protein that controls the function of HIPK2. Here we show that DCAF7 does not bind to HIPK1, although HIPK2 and HIPK1 share a common domain structure and a high degree of sequence similarity^29^. In stark contrast, HIPK2 is not structurally related with DYRKs outside the catalytic domain. Nevertheless, we identified a functional DCAF7 binding site in the N-terminal region of HIPK2. This sequence is fully conserved in HIPK2 from vertebrates, but is absent from HIPK orthologs from lower organisms. Thus, HIPK2 may have acquired the capacity to bind DCAF7 by convergent evolution after the separation of the HIPK1 and HIPK2 genes. DCAF7 controls the threshold, amplitude, and kinetics of HIPK2-triggered signalling events^18^, suggesting that the association with DCAF7 is a functionally critical difference between HIPK1 and HIPK2^29^.

Given that DCAF7 binds to HIPK2 in a manner similar to DYRK1A, it is not surprising that the HIPK2/DCAF7 complex was also recruited to E1A. Interestingly, HIPK2 has previously been identified as a hit in a yeast two hybrid screen in which a complex of E1A and CtBP (C-terminal-binding protein) was used as the bait^47^. The authors of this study propose that HIPK2 directly binds to CtBP, rather than E1A, whereas our results show that E1A interacts with HIPK2 *via* DCAF7. Further work will be necessary to exactly define the roles of CtBP and DCAF7 in the interaction of E1A and HIPK2.

The deletion of the DCAF7 interaction site in E1A results in a hypertransforming effect of the adenoviral oncoprotein^15^. This observation points to an important role of interaction with DYRK1A or HIPK2 on cellular growth. Further experiments will be necessary to reveal the individual contributions of these kinases on the biological effects of E1A.

## Materials and Methods

### Antibodies

The following commercially available antibodies were used: rabbit monoclonal anti-DCAF7 (EPR8712, Epitomics/Abcam), rabbit polyclonal anti GFP (Clontech 632592), goat polyclonal anti-GFP (Rockland) and anti-c-Myc (Santa Cruz Biotechnology), mouse monoclonal anti-DYRK1A (clone 7D10, Abnova, directed against a C-terminal epitope), anti FLAG (clone M2, Sigma), anti HA (clone 3F10, Roche) and anti E1A-pSer219 (C-9, sc-374663, Santa Cruz Biotechnology). Monoclonal mouse antibodies for the adenovirus E1A protein (M58 and M73 hybridoma supernatants were produced as described^48^ and anti-HA clone 12CA5 was a gift from Dr. Fred Dick). The M58 anti E1A and M2 anti FLAG (F-1804, Sigma) monoclonal antibodies were used for immunofluorescence detection. A custom-made rabbit antiserum directed against DYRK1B^49^ and a custom-made polyclonal goat antibody directed against an N-terminal epitope of DYRK1A (PHSHQYSDRRQPN, Everest Biotech, UK) were used for IP.

### Plasmids

Bacterial and mammalian expression vectors are described in the Supplementary Methods.

### Cell culture and transfection

HeLa cells were grown using Quantum 101 complete medium for HeLa cells (PAA) or RPMI 1640 (PAA) with 10% fetal bovine serum and kept at 37 °C in a humidified 5% CO_2_ atmosphere. Human fibrosarcoma HT-1080 cells and HEK293 cells were kept in DMEM (Multicell) or DMEM high glucose (Sigma), respectively, supplemented with 10% fetal bovine serum. HEK293 cells and HeLa cells were transiently transfected using the FuGENE HD reagent (Promega) either in suspension before the cells were plated or on the following day after medium change. HT1080 cells were transfected using X-tremeGeneHP reagent (Roche) following the manufacturer’s recommendations. HEK293-(GFP-DYRK1A-tetOn) cells^50^ were treated for 24 h with 2 μg/mL doxycyclin to induce GFP-DYRK1A expression.

### Immunoprecipitation and Western blotting

Cells were washed twice with cold PBS and then lysed on ice for 20 minutes in 1 mL non-denaturing lysis buffer (50 mM Tris pH 7.4, 150 mM NaCl, 15% glycerol, 1 mM EDTA, 1 mM NaF, 0.5% Igepal CA 630) supplemented with phosphatase-/protease inhibitors (1 mM Na_3_VO_4_, 2.5 μg/μL aprotinin, 1 μg/μL pepstatin A, 10 μg/μL leupeptin, 100 mM phenylmethanesulfonyl fluoride). Lysates were sonicated twice for 45 s and cleared by centrifugation (14,000 rpm for 3 min at 4 °C). Aliquots were taken for the input control. For the IP of GFP fusion proteins, extracts were tumbled for 1 h at 4 °C with 10 μL of paramagnetic GFP-Trap beads (Chromotek). The immunoprecipitates were isolated using a magnetic rack and washed 3 times (50 mM Tris pH 8.0, 150 mM NaCl, 2 mM EDTA pH 8.0, 0.1% Igepal CA 630). Bound proteins were eluted in Laemmli’s sample buffer for SDS-PAGE and Western blot analysis. All other IPs were performed overnight at 4 °C using anti-FLAG M2 affinity gel (Sigma) or custom-made antibodies and protein G-coupled sepharose (Sigma-Aldrich). Beads were washed and eluted with gel loading buffer for SDS-PAGE. Native elution from FLAG M2 affinity gel was achieved by incubation with an excess of 3xFLAG peptide (Sigma) at 4 °C for 30 min at a final concentration of 200 μg/mL. Samples were resolved on 8%, 10% or 12% acrylamide gels, depending on the size of the relevant protein, and transferred onto nitrocellulose membrane. Western blots were developed using horseradish peroxidase-coupled secondary antibodies (Rockland). The Clean-Blot Detection Reagent (Thermo Scientific) was used as a secondary antibody for the detection of proteins that had been precipitated with the same antibody. Chemiluminescence signals were recorded using a LAS-3000 CCD imaging system (Fujifilm) and the AIDA 3.52 software (Raytest).

### RNAi knockdown

Downregulation of DYRK1A and DCAF7 were performed using Silencer Select siRNA (Ambion, DCAF7 id: s19985, DYRK1A id: s4400) in HT1080 cells, because this cell line gave the best knockdown effect. siRNA was delivered to HT1080 cells at a final concentration of 10 nM via transfection with siLentFect (BioRad) following the manufacturer’s instructions for a period of 24 h. A scrambled siRNA was used as a control. Ensuing co-IP and western blot analysis were performed as described previously^51^.

### Bacterial expression of recombinant proteins

GST fusion proteins were expressed in *E. coli* and affinity purified using glutathione Sepharose 4B (Amersham plc). After extensive washing, the recombinant proteins were either eluted with 10 mM reduced glutathione in 50 mM Tris-HCl (pH 8.0) for use in kinase assays, or the resin with the bound protein was stored at −70 °C as a suspension in 50% glycerol/phosphate buffered saline for use in pulldown assays. Aliquots of the samples were analysed side by side with known amounts of bovine serum albumin by SDS-PAGE and subsequent Coomassie staining to adjust the amounts of protein used in the assays.

### GST-pulldown from HeLa cells

HeLa cell lysates were processed from one near confluent 9 cm-dish as described above. Extracts were split in 2–3 aliquots and GST-tagged proteins immobilized on glutathione-Sepharose (5 μg of GST protein per sample) were added as indicated in the figure legends. Samples were incubated for 2 h at 4 °C in a buffer containing 10 mM Tris pH 8.0, 150 mM NaCl, 0.5 mM EDTA, 0.05% Igepal CA-630 supplemented with protease and phosphatase inhibitors (end-over-end rotation), followed by extensive washing. Bound proteins were eluted from the beads in Laemmli’s sample buffer for 5 min at 95 °C before SDS-PAGE and western blot analysis.

### *In vitro*-interaction assays

*In vitro*-transcription of the mRNA template was performed using the pLEXSY-invitro-2 vector (Suppl. Methods) and a T7 RNA polymerase-dependent kit (Thermo Scientific). The mRNA was used without purification to drive translation of GFP-hDCAF7 in the rabbit reticulocyte translation system (Promega) for 1.5 h at 37 °C followed by 2 h at 30 °C. For GST-pulldown assays, 10 μL of the reaction mix were incubated for 2 h at 4 °C with ~10 μg of GST fusion proteins immobilized to glutathione Sepharose in a volume of 600 μL. The bait proteins were eluted with an excess of glutathione (10 mM) and bound proteins were detected by Western blotting. To produce untagged E1A-X2, glutathione Sepharose resin with immobilized GST-E1A-X2 was incubated with human thrombin (MP Biomedicals) for 3 h at 22 °C. The reaction was stopped by adding PMSF to a final concentration of 1 mM and the supernatant was used for interaction assays without further purification (Fig. S4).

### E1A phosphorylation *in vitro* and in living cells

For *in vitro* kinase assays, GST-E1A-X2 (0.1 μg/μL) was incubated at 30 °C in kinase buffer (25 mM Hepes pH 7.4, 0.5 mM dithiothreitol, 5 mM MgCl_2_, 1 mM Na_3_VO_4_) and 1 mM ATP. The reaction was started by addition of GST-DYRK1A-ΔC (10 ng/μL). This mutant is routinely used for kinase assays because it exhibits the same substrate specificity but has higher specific activity than the wild type kinase^31^,^32^. Aliquot of 10 μL were taken at variable times and the reaction was stopped by addition of 2 μL of 100 mM EDTA. Phosphorylation of E1A was detected by Western blot analysis with a phosphospecific antibody directed against pSer219. To detect the phosphorylation of endogenous E1A (in HEK293 cells) or overexpressed myc-E1A (in HeLa cells), cells were grown in 6-well plates and transfected as indicated. Cells were lysed with hot SDS lysis buffer (20 mM Tris pH 7.4, 1% SDS, 100 μL/well) and total cellular lysates were analysed for E1A phosphorylation by western blotting.

### Immunofluorescence microscopy

HeLa cells were grown on glass coverslips in 24-well plates in DMEM (10% fetal bovine serum) at 37 °C and transfected with plasmids as indicated in the figure legend using X-tremeGENE HP (Roche). Twenty-four hours later, cells were fixed in 3.7% paraformaldehyde, permeabilized on ice using 0.2% Triton X-100, and blocked with 3% bovine serum albumin in PBS. Samples were successively incubated with primary and secondary antibodies (1:300, goat anti-mouse Alexa Fluor 594; Life Technologies) each for 1 h at room temperature and mounted using ProLong Gold reagent containing DAPI (4′,6-diamidino-2-phenylindole). Images were acquired using a Fluoview 1000 laser scanning confocal microscope (Olympus).

## Additional Information

**How to cite this article**: Glenewinkel, F. *et al.* The adaptor protein DCAF7 mediates the interaction of the adenovirus E1A oncoprotein with the protein kinases DYRK1A and HIPK2. *Sci. Rep.* **6**, 28241; doi: 10.1038/srep28241 (2016).

## Supplementary Material

Supplementary Information
