# Supplementary material for: The adaptor protein DCAF7 mediates the interaction of the adenovirus E1A oncoprotein with the protein kinases DYRK1A and HIPK2
Source: Sci Rep. 2016 Jun 16;6:28241. doi: 10.1038/srep28241 (PMC4910162; doi:10.1038/srep28241)
Supplement: Supplementary Information [file srep28241-s1.pdf]

Supplementary Material

The adaptor protein DCAF7 mediates the interaction of the adenovirus E1A oncoprotein with the protein kinases DYRK1A and HIPK2

Florian Glenewinkel[1], Michael J. Cohen[2], Cason R. King[2], Sophie Kaspar[1], Simone Bamberg-Lemper[1], Joe S. Mymryk[2], Walter Becker[1*]

1 Institute of Pharmacology and Toxicology, RWTH Aachen University, Wendlingweg 2, 52074 Aachen, Germany
2 Departments of Microbiology & Immunology and Oncology, University of Western Ontario, London, Ontario, Canada

Supplementary figures:

Fig. S1: Nuclear localization of wild type GFP-DYRK1A and GFP-DYRK1A-Δ93-104

Fig. S2: Mapping of the DCAF7-interacting sequence in DYRK1A

Fig. S3: Co-IP of endogenous E1A and DCAF7 with GFP-HIPK2

Fig. S4: *In vitro* assembly of the DYRK1A/DCAF7/E1A complex

Fig. S5: Co-IP of myc-E1A with GFP-DYRK1A deletion constructs

Fig. S6: Pulldown of DYRK1A deletion constructs by GST-E1A(X2)

Fig. S7: Phosphorylation of E1A(X2) by DYRK1A and HIPK2 in HeLa cells

Supplementary methods:

Cloning of *Dictyostelium* DYRK1

Vector for *in vitro* transcription and subsequent *in vitro* translation of DCAF7

Mammalian expression vectors for E1A

Sources of previously described plasmids

Supplementary Figures

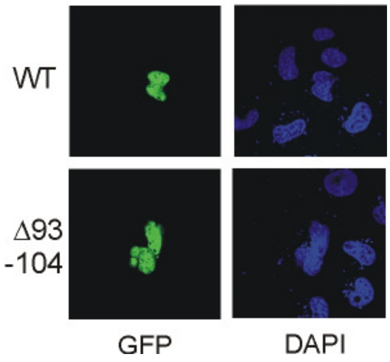

Figure S1: Nuclear localization of wild type GFP-DYRK1A and GFP-DYRK1A-Δ93-104
HeLa cells were transiently transfected to express wild type GFP-DYRK1A (WT) or GFP-DYRK1A-Δ93-104. GFP fusion proteins were detected by autofluorescence (GFP) and nuclei were stained with DAPI.

A

```
DYRK1A   77 LQRRMPQTFRDPATAPLRKLSVDLIKTY 104
Δ93-104  77 LQRRMPQTFRDPATAPL----------- 104
LRK→A    77 LQRRMPQTFRDPATAPAAALSVDLIKTY 104
LSVD→A   77 LQRRMPQTFRDPATAPLRKAAAALIKTY 104
LIKT→A   77 LQRRMPQTFRDPATAPLRKLSVDAAAAY 104
```

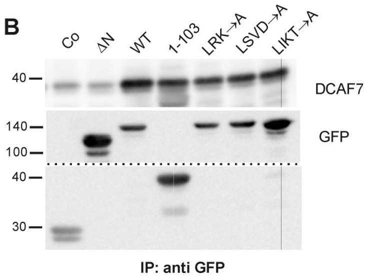

Figure S2: Mapping of the DCAF7-interacting sequence in DYRK1A

A) Multiple residues in the DCAF7 binding region were mutated to define the contribution of these residues. The deletion mutant defective in DCAF7 binding (Δ93-104, see Fig. 2) is shown for comparison.

B) Co-IP of FLAG-DCAF7 with GFP-DYRK1A mutants. GFP-DYRK1A$_{1\text{-}103}$ served as a positive control and GFP-DYRK1A-ΔN was used as a negative control.

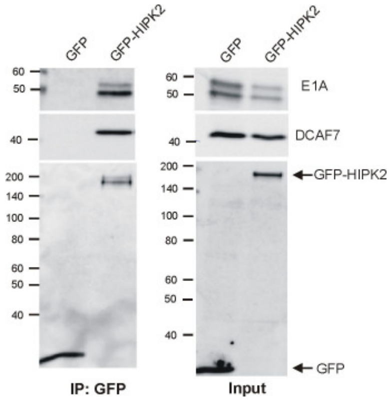

Figure S3: Co-IP of endogenous E1A and DCAF7 with GFP-HIPK2.
HEK293 cells were transfected to overexpress GFP-HIPK2 or GFP and subjected to anti GFP immunoprecipitation. Bound proteins were detected by immunoblotting using antibodies directed against DCAF7, E1A and GFP.

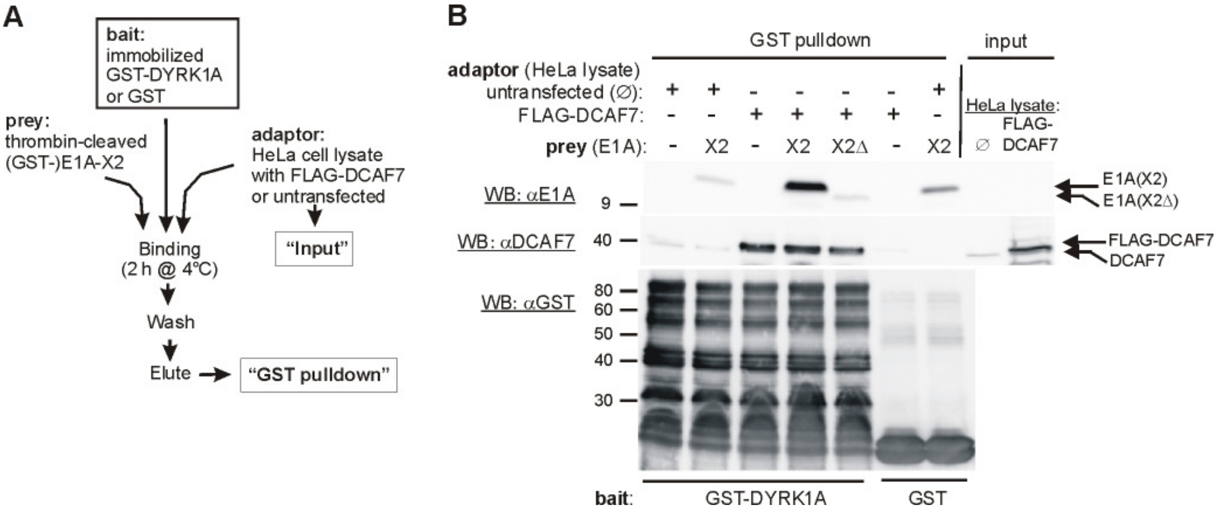

Figure S4: *In vitro* assembly of the DYRK1A/DCAF7/E1A complex

A) Outline of the experiment. The vector-encoded thrombin cleavage site was used to produce untagged prey protein from bacterially expressed GST-E1A-X2 or GST-E1A-X2Δ. Cell lysates of transiently transfected HeLa cells were used as the source for FLAG-DCAF7 (adaptor). The pulldown experiment was performed with bacterially expressed GST-DYRK1A that was immobilized to glutathione Sepharose as in Fig. 4e.

B) Western blot analysis. Binding of E1A-X2 to GST-DYRK1A depends on the presence of FLAG-DCAF7. Direct binding of E1A-X2 to GST-DYRK1A (second lane) does not exceed the non-specific background as revealed by pulldown with GST.

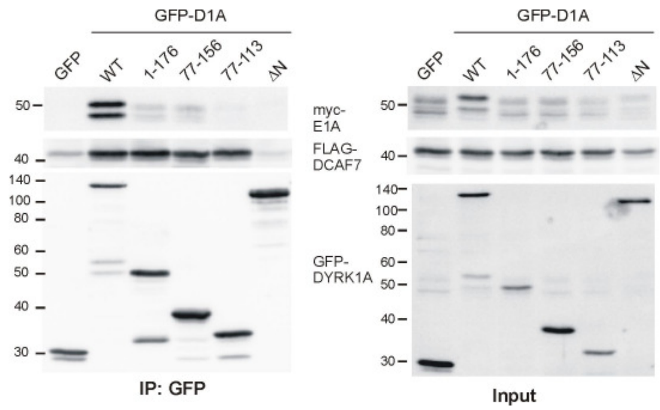

Figure S5: Co-IP of myc-E1A with GFP-DYRK1A deletion constructs

HeLa cells co-expressing myc-E1A, FLAG-DCAF7 and the indicated GFP-DYRK1A constructs were used for anti GFP IP. The recombinant proteins were detected be immunoblotting with antibodies directed against GFP, DCAF7 or the myc epitope.

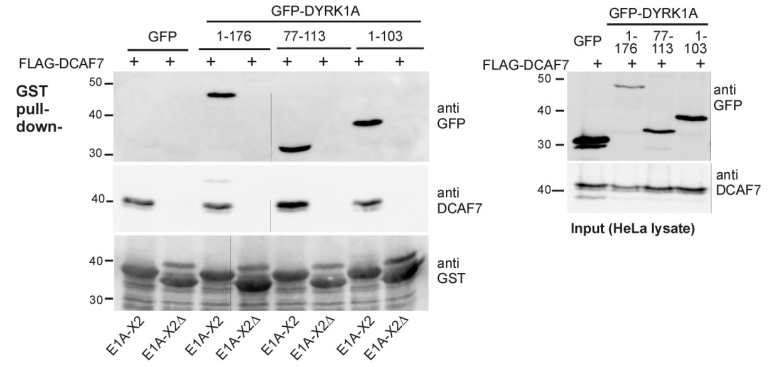

Figure S6: Pulldown of DYRK1A deletion constructs by GST-E1A-X2

HeLa cells were transfected to co-express FLAG-DCAF7 with GFP-DYRK1A deletion constructs as indicated. Cell lysates were subjected to GST-pulldown assay with immobilized GST-E1A-X2 or GST-E1A-X2Δ and bound proteins were analysed by immunoblotting.

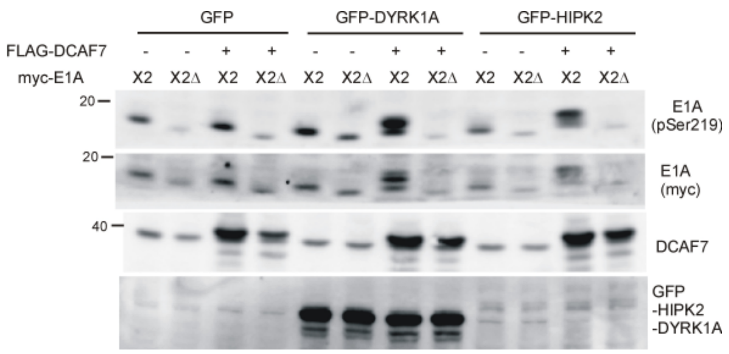

Figure S7: Phosphorylation of E1A(X2) by DYRK1A and HIPK2 in HeLa cells

HeLa cells were co-transfected with expression plasmids for myc-E1A-X2 or myc-E1A-X2Δ, FLAG-DCAF7 and GFP-DYRK1A, GFP-HIPK2 or empty GFP vector as indicated. Two days after transfection, Western blots of total lysates were analysed for phosphorylation of E1A. E1A-X2Δ is a deletion mutant lacking the DCAF7 binding region (deletion of amino acids 255-270).

Supplementary Methods:

Cloning of *Dictyostelium* DYRK1

To generate a mammalian expression vector for *Dictyostelium discoideum* DYRK1 (Uniprot Q76NV1), the segment of the gene encoding amino acids 1-40 was amplified from genomic DNA (kindly provided by Annette Müller-Taubenberger, Ludwig Maximilian University of München, Germany) and cloned into pEGFP-C1 using the following primers with engineered restriction sites*:*

ddDYRK1Afor	atggat**AGATCT**gcaaaatcaagaaatgatgcacc (*BglII*)
ddDYRK1Arev	gttgtt**AAGCTT**attgctttttttttgctttggcagtg (*HindIII*)

Vector for *in vitro* transcription and subsequent *in vitro* translation of DCAF7

The coding sequence of hDCAF7 was inserted C-terminal of the reading frame for GFP in the vector pLEXSY_invitro_2 (Jena Bioscience, Jena, Germany) *via* engineered restriction sites in the PCR primers:

DCAF7for	acgac**AAGCTT**ctatgtccctgcacggc *(HindIII)*
DCAF7rev	atat**GCGGCCGC**ctacactctgagtatctccagg *(NotI)*

Mammalian expression vector for E1A

To construct vectors for mammalian expression of myc-E1A (289 amino acid form), myc-E1A-X2 (containing only exon 2) and the deletion mutant myc-E1A(X2Δ) (deletion of amino acids 255-270), inserts were ligated in frame with the myc epitope tag present in pCANmyc.

Previously described plasmids

All other expression vectors and their mutated versions are listed in table S1.

Table S1: Sources of the expression plasmids

Expressed protein	Species	Variants	Reference
GFP-rDYRK1A	rat	WT, 1-103, 1-176	Becker et al. 1998
GFP-rDYRK1A	rat	K188R	Himpel et al. 2001
GFP-rDYRK1A	rat	Δ1-135, Δ93-104, 77-158, 77-113, 77-136, alanine mutants	this work
FLAG-mDYRK1A	mouse	WT	Sitz et al. 2008
HA-rDYRK1A	rat	WT	Kentrup et al. 1996
GST-DYRK1A	rat	WT, ΔC	Himpel et al. 2001
GFP-hDYRK1B	human	WT (p69)	Leder et al. 2003
hDYRK1B	human	WT (p69)	Leder et al. 2003
xDYRK1B	*Xenopus laevis*	WT	Lilienthal et al. 2010
GFP-hHIPK1	mouse	WT	Kim et al. 1998
GFP-hHIPK2	human	WT	Hofmann et al. 2002
GFP-hHIPK2	human	D243N	van der Laden et al 2015
GFP-hHIPK2	human	T125P, 1-114, 1-135	this work
GFP-zDCAF7	zebrafish	WT	Nissen et al. 2006
FLAG-hDCAF7	human	WT	Ritterhoff et al. 2010
GFP-E1A	HAdV-5	WT, R262/263E	Cohen et al. 2013
GST-E1A-X2	HAdV-5	WT, Δ255-270	Avvakumov et al. 2002
E1A	HAdV-5	12S	Rasti et al. 2005

References

Avvakumov, N., Sahbegovic, M., Zhang, Z., Shuen, M. & Mymryk, J.S. Analysis of DNA binding by the adenovirus type 5 E1A oncoprotein. *J. Gen. Virol.* **83**, 517-24 (2002).

Becker, W., Weber, Y., Wetzel, K., Eirmbter, K., Tejedor, F.J. & Joost, H.G. Sequence characteristics, subcellular localization, and substrate specificity of DYRK-related kinases, a novel family of dual specificity protein kinases. *J. Biol. Chem.* **273**, 25893-902 (1998).

Cohen, M.J., Yousef, A.F., Massimi, P., Fonseca, G.J., Todorovic, B., Pelka, P., Turnell, A.S., Banks, L. & Mymryk, J.S. Dissection of the C-terminal region of E1A redefines the roles of CtBP and other cellular targets in oncogenic transformation. *J. Virol*. **87**, 10348-55 (2013).

Himpel, S., Panzer, P., Eirmbter, K., Czajkowska, H., Sayed, M., Packman, L.C., Blundell, T., Kentrup, H., Grötzinger, J., Joost, H.G. & Becker, W. Identification of the autophosphorylation sites and characterization of their effects in the protein kinase DYRK1A. *Biochem. J.* **359**, 497–505 (2001).

Hofmann, T.G., Möller, A., Sirma, H., Zentgraf, H., Taya, Y., Dröge, W., Will, H. & Schmitz, M.L. Regulation of p53 activity by its interaction with homeodomain-interacting protein kinase-2. *Nat. Cell Biol.* **4**, 1–10 (2002).

Kentrup, H., Becker, W., Heukelbach, J., Wilmes, A., Schürmann, A., Huppertz, C., Kainulainen, H. & Joost, H.G. Dyrk, a dual specificity protein kinase with unique structural features whose activity is dependent on tyrosine residues between subdomains VII and VIII. *J. Biol. Chem.* **271**, 3488-95 (1996).

Kim, Y.H., Choi, C.Y., Lee, S.J., Conti, M.A. & Kim, Y. Homeodomain-interacting protein kinases, a novel family of co-repressors for homeodomain transcription factors. *J Biol Chem*. **273**, 25875–9 (1998).

Leder, S., Czajkowska, H., Maenz, B., de Graaf, K., Barthel, A., Joost, H.G. & Becker, W. Alternative splicing variants of dual specificity tyrosine phosphorylated and regulated kinase 1B exhibit distinct patterns of expression and functional properties. *Biochem. J.* **372**, 881-8 (2003).

Lilienthal, E., Kolanowski, K. & Becker, W. Development of a sensitive non-radioactive protein kinase assay and its application for detecting DYRK activity in Xenopus laevis oocytes. *BMC Biochem.* **11**, 20 (2010).

Nissen, R.M., Amsterdam, A. & Hopkins, N. A zebrafish screen for craniofacial mutants identifies wdr68 as a highly conserved gene required for endothelin-1 expression. *BMC Dev. Biol.* **6**, 28 (2006).

Rasti, M., Grand, R.J., Mymryk, J.S., Gallimore, P.H. & Turnell, A.S. Recruitment of CBP/p300, TATA-binding protein, and S8 to distinct regions at the N terminus of adenovirus E1A. *J. Virol.* **79,** 5594-605 (2005).

Ritterhoff, S., Farah, C.M., Grabitzki, J., Lochnit, G., Skurat, A.V. & Schmitz, M.L. The WD40-repeat protein Han11 functions as a scaffold protein to control HIPK2 and MEKK1 kinase functions. *EMBO J*. **29,** 3750-61 (2010).

Sitz, J.H., Baumgärtel, K., Hämmerle, B., Papadopoulos, C., Hekerman, P., Tejedor, F.J., Becker, W.& Lutz, B. The Down syndrome candidate dual-specificity tyrosine phosphorylation-regulated kinase 1A phosphorylates the neurodegeneration-related septin 4. *Neuroscience* **157**, 596-605 (2008).

van der Laden, J., Soppa, U. & Becker W. Effect of tyrosine autophosphorylation on catalytic activity and subcellular localisation of homeodomain-interacting protein kinases (HIPK). *Cell Commun. Signal.* **13,** 3 (2015).
